# Caveolin Contributes to the Modulation of Basal and β-Adrenoceptor Stimulated Function of the Adult Rat Ventricular Myocyte by Simvastatin: A Novel Pleiotropic Effect

**DOI:** 10.1371/journal.pone.0106905

**Published:** 2014-09-11

**Authors:** Sara D. Pugh, David A. MacDougall, Shailesh R. Agarwal, Robert D. Harvey, Karen E. Porter, Sarah Calaghan

**Affiliations:** 1 School of Biomedical Sciences, University of Leeds, Leeds, West Yorkshire, United Kingdom; 2 Division of Cardiovascular and Diabetes Research, University of Leeds, Leeds, West Yorkshire, United Kingdom; 3 Department of Pharmacology, University of Nevada Reno, Reno, Nevada, United States of America; University of Hull, United Kingdom

## Abstract

The number of people taking statins is increasing across the globe, highlighting the importance of fully understanding statins' effects on the cardiovascular system. The beneficial impact of statins extends well beyond regression of atherosclerosis to include direct effects on tissues of the cardiovascular system (‘pleiotropic effects’). Pleiotropic effects on the cardiac myocyte are often overlooked. Here we consider the contribution of the caveolin protein, whose expression and cellular distribution is dependent on cholesterol, to statin effects on the cardiac myocyte. Caveolin is a structural and regulatory component of caveolae, and is a key regulator of cardiac contractile function and adrenergic responsiveness. We employed an experimental model in which inhibition of myocyte HMG CoA reductase could be studied in the absence of paracrine influences from non-myocyte cells. Adult rat ventricular myocytes were treated with 10 µM simvastatin for 2 days. Simvastatin treatment reduced myocyte cholesterol, caveolin 3 and caveolar density. Negative inotropic and positive lusitropic effects (with corresponding changes in [Ca^2+^]_i_) were seen in statin-treated cells. Simvastatin significantly potentiated the inotropic response to β2-, but not β1-, adrenoceptor stimulation. Under conditions of β2-adrenoceptor stimulation, phosphorylation of phospholamban at Ser^16^ and troponin I at Ser^23/24^ was enhanced with statin treatment. Simvastatin increased NO production without significant effects on eNOS expression or phosphorylation (Ser^1177^), consistent with the reduced expression of caveolin 3, its constitutive inhibitor. In conclusion, statin treatment can reduce caveolin 3 expression, with functional consequences consistent with the known role of caveolae in the cardiac cell. These data are likely to be of significance, particularly during the early phases of statin treatment, and in patients with heart failure who have altered β-adrenoceptor signalling. In addition, as caveolin is ubiquitously expressed and has myriad tissue-specific functions, the impact of statin-dependent changes in caveolin is likely to have many other functional sequelae.

## Introduction

The number of people taking statins will increase as the threshold for statin prescription in cardiovascular disease prevention continues to decrease across the globe. For example, recent amendments to ACC/AHA guidelines for statin prescription [1] mean that 13 million more individuals, and a total of 49% of adults between 40 and 75, are now eligible for statin therapy in the US [2]. This, combined with high-dose statin treatment regimes recommended for some patient groups (e.g. [3]), highlights the importance of fully understanding statins' effects on the cardiovascular system. Clinical and experimental data support the view that the impact of statins on the cardiovascular system extends well beyond lowering serum LDL cholesterol; many of their beneficial effects can be ascribed to direct actions on tissues of the cardiovascular system (‘pleiotropic’ effects). Improved endothelial function and reduced vascular smooth muscle proliferation, fibrosis, platelet aggregation are just a few of the many pleiotropic changes which statins have been shown to elicit [4]–[7]. Surprisingly, little work has focused on direct effects of statins on the cardiac myocyte.

Pleiotropic effects of statins have often been linked with depletion of isoprenoid intermediates of the HMG CoA reductase pathway, particularly farnesyl pyrophosphate (FPP) and geranylgeranyl pyrophosphate (GGPP) which modulate signalling by facilitating membrane targeting of elements including small and heterotrimeric G proteins. Endothelial dysfunction, characterised by reduced bioavailability of NO, is a common finding in cardiovascular disease and increased NO production with statin treatment provides an excellent illustration of the breadth of statins' pleiotropic effects. Statins alter NO bioavailability by a number of different isoprenoid-dependent mechanisms including increased eNOS expression (via Rho stabilisation of eNOS mRNA [8]), enhanced eNOS activity (via Ras-dependent Akt phosphorylation [9]), reduced NO scavenging (via Rac1-dependent effects on NADPH oxidase activity [10]).

However, in jumping on the ‘isoprenoid bandwagon’, one factor which is often overlooked is that alterations in *tissue* cholesterol may also make a significant contribution to statins' pleiotropic effects. Statins may initially alter tissue cholesterol levels by limiting *de novo* cholesterol synthesis. Nevertheless, cellular cholesterol homeostasis is maintained through transcriptional regulation, via sterol regulatory elements (SRE), of proteins involved in the synthesis (HMG CoA reductase), uptake (LDL receptors), and efflux (scavenger receptor class B type I, ABC transporters, caveolin) of cholesterol [11]. Whilst any changes in tissue cholesterol are likely to be normalised to some extent by transcriptional control, altered expression of cholesterol homeostatic proteins may have functional consequences. Certainly this is the case for NO bioavailability. Statin treatment has been shown to decrease the expression of caveolin 1 in endothelial cells and, as caveolin is a key inhibitory regulator of eNOS, thereby increase eNOS activity [12].

As well as its contribution to cholesterol homeostasis, caveolin is of fundamental importance in most cells as a structural component of caveolae, invaginated lipid rafts, and as a regulatory scaffold [13]. Caveolae are required for normal cardiac function: caveolin 3 (Cav3) is the muscle-specific caveolin isoform and Cav3 knockout mice develop hypertrophy and cardiomyopathy [14]. Both decreased and increased expression of Cav3 have detrimental effects on the heart, illustrating the need to maintain caveolin expression within strict limits for optimal cardiac function [14], [15]. Acute manipulation of caveolae/caveolin binding has demonstrated that cardiac myocyte contractile function and β adrenoceptor (AR) responsiveness are exquisitely dependent on caveolae and caveolin [16]–[19]. Given all this, the potential impact of statin treatment on myocyte contractility or the response to sympathetic stimulation is an important consideration, particularly in patients with heart failure. Here we address this gap in knowledge by examining the effect of simvastatin treatment on adult rat ventricular myocyte function. Simvastatin and atorvastatin are the most widely used statins because the patents for these drugs have expired, dramatically reducing the cost of prescription. We chose simvastatin for our study because its patent expired a decade before that of atorvastatin (in 2003 in the UK). Our priority was to use cells which provide a true structural and functional representation of myocytes in the human heart (i.e. adult ventricular myocytes). We used a simple model system in which effects of HMG CoA reductase inhibition on the ventricular myocyte could be studied in the absence of alterations in cholesterol influx/efflux or paracrine influences from other cells in the myocardium.

This work shows for the first time that inhibition of cholesterol synthesis has the capacity to modestly depress basal cardiac inotropy and enhance lusitropy, whilst increasing β-AR responsiveness. Our data suggest that these functional changes are due in part to altered caveolin expression and distribution. However, a contribution from isoprenoid-dependent effects cannot be excluded.

## Results

### Simvastatin reduces cellular and caveolar cholesterol

**Figure 1:**
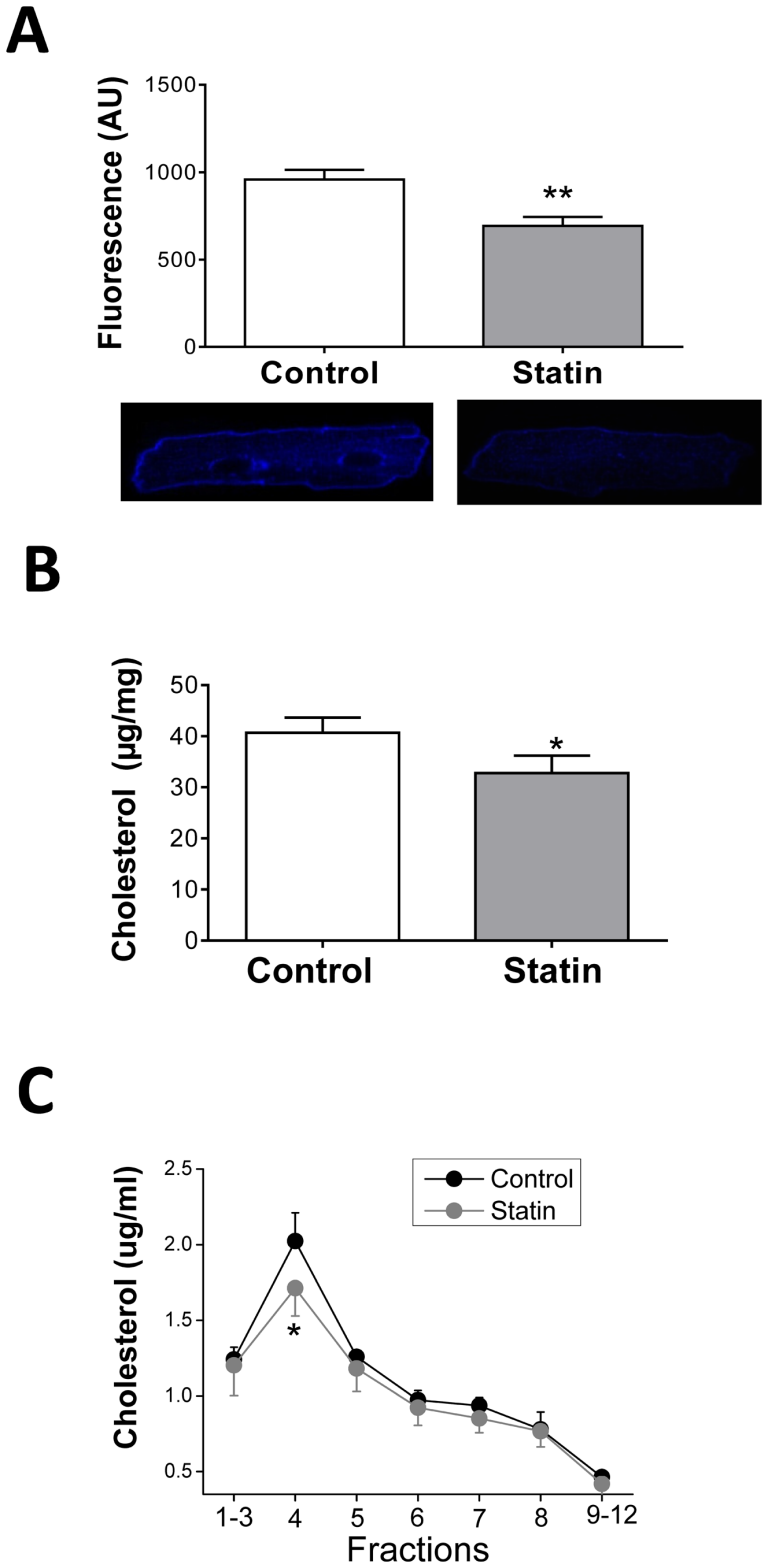
Treatment with 10 µM simvastatin for 48 h reduces cellular and caveolar cholesterol in the adult ventricular myocyte. A. Cellular free cholesterol indexed in fixed myocytes labelled with filipin. Bar graph shows mean fluorescence (arbitrary units, AU) from n = 48-58 myocytes (from 4 hearts). Representative control and statin-treated myocytes are shown below. B. Total cholesterol (normalised to total protein concentration) measured in cell lysates using the Amplex Red assay (n = 7 hearts). C. Cholesterol distribution measured in cell lysates following sucrose density gradient fractionation using the Amplex Red assay. Samples were adjusted to equal protein concentrations before fractionation (n = 3 hearts). All data are mean ± S.E.M. * P<0.05, ** P<0.01 vs. control, Student's t-test.

Our study employed a simple experimental model to look directly at the acute effects of statin treatment on the adult cardiac myocyte. Our first question was whether culturing myocytes in the presence of simvastatin reduces cellular cholesterol i.e. does simvastatin reduce *de novo* myocyte cholesterol synthesis? In order to select a concentration of simvastatin which is of clinical relevance, we considered serum levels of drug, tissue accumulation and IC_50_. In man, an oral dose of 40 mg simvastatin gives peak serum levels of up to 0.1 µM [20], yet therapeutic regimes of high dose simvastatin (up to 80 mg/day), lead to accumulation in tissue at levels in excess of this (e.g. 12 µM in stomach, 1 µM in spleen and testis), because of the lipophilic nature of the drug [21], [22]. The IC_50_ of simvastatin for HMG CoA reductase is 0.1 µM in rat hepatocytes [23]. In preliminary studies we compared the effect of 1 µM and 10 µM simvastatin treatment on myocyte free cholesterol indexed using the fluorescent antibiotic filipin. With 1 µM simvastatin cholesterol was significantly reduced (P<0.05) by 31% after 5 days. A similar level of cholesterol depletion was achieved by a 2 day treatment with 10 µM simvastatin (by 28%, P<0.05). In order to minimise genotypic and phenotypic changes in the adult myocyte which are known to occur in culture and can have profound effects on myocyte contractility [24], we elected to study the effects of 10 µM simvastatin after 2 days in all subsequent experiments. Depletion of cholesterol was apparent throughout the cell, in both surface membrane and deeper regions (which include t-tubules) (Fig. 1A). We also assessed total cholesterol in cell lysates using the Amplex Red assay and saw a similar degree of cholesterol depletion (P<0.05) in statin-treated preparations (Fig. 1B). Following fractionation of cell homogenates on a sucrose density gradient, a selective reduction in cholesterol in buoyant raft/caveolar fraction 4 was observed (P<0.05; Fig. 1C). High levels of cholesterol in this fraction may facilitate detection of changes in cholesterol; we do not exclude the possibility that simvastatin induces small changes in cholesterol in other fractions of the cell.

### Simvastatin reduces cellular and caveolar Cav3

**Figure 2:**
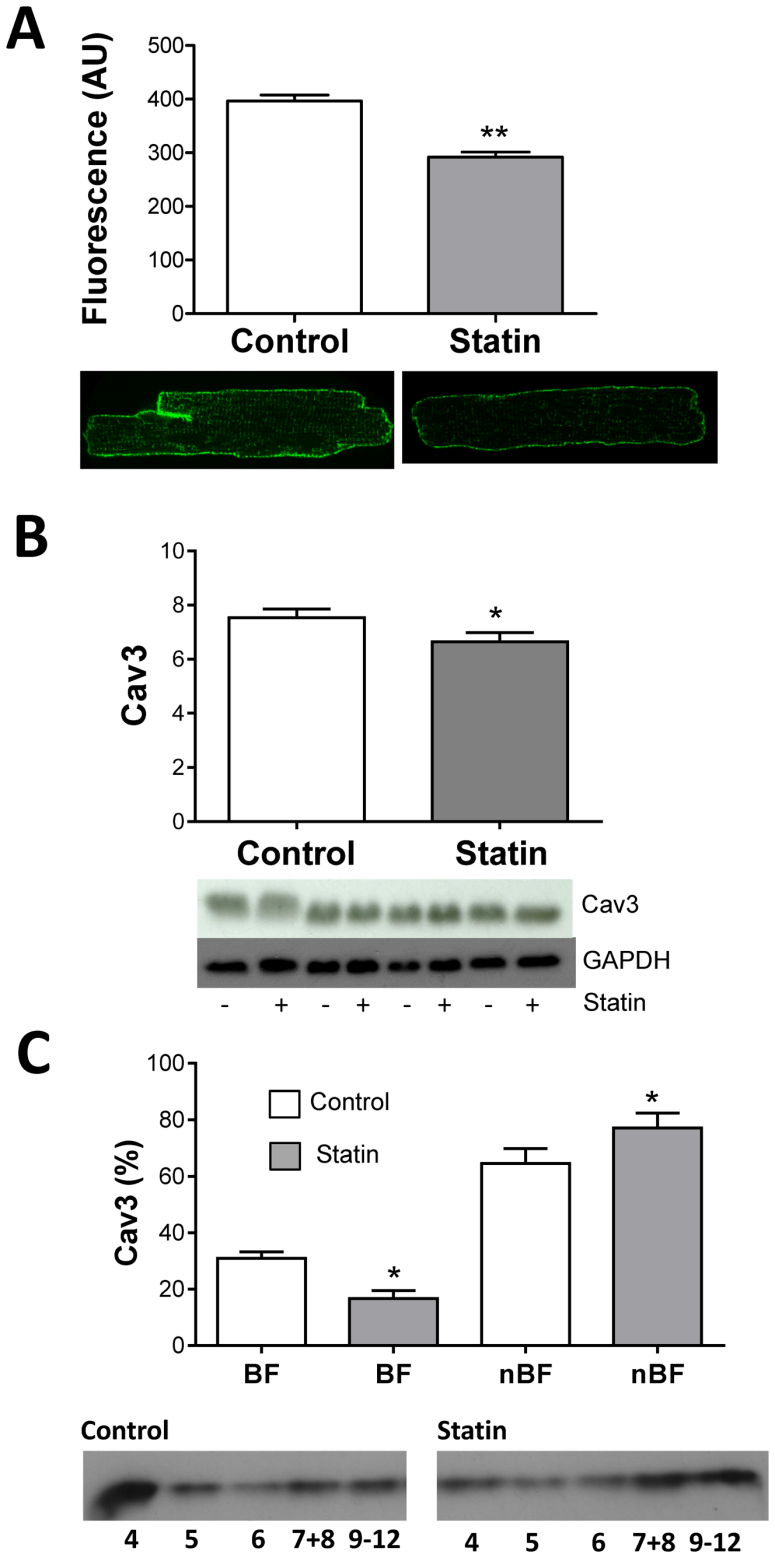
Simvastatin treatment reduces cellular and caveolar Cav3. A. Total membrane-bound Cav3 indexed using immunocytochemistry. Bar graph shows mean fluorescence (arbitrary units, AU) from 59–70 myocytes. Representative control and statin-treated myocytes are shown below. B. Total cellular Cav3 measured in cell lysates using Western blotting. Values are normalised to GAPDH. C. Membrane distribution of Cav3 measured in cell lysates following sucrose density gradient fractionation using Western blotting. Fractions 4 and 5 are cholesterol-enriched buoyant fractions (BF); fractions 7–12 are non-buoyant fractions (nBF). All data are mean + S.E.M from n = 3–4 hearts. * P<0.05, ** P<0.01 vs. control, Student's t-test.

Next we addressed the question of whether simvastatin affects the expression of Cav3 - the muscle-specific caveolin isoform. Cav3 staining in fixed permeabilised cells was reduced by ≈30% (P<0.01) following statin treatment (Fig. 2A); depletion of Cav3 was apparent in both surface and t-tubular membranes. In cell lysates, total Cav3 expression was also reduced (P<0.05) (Fig. 2B). These data support the view that Cav3 expression may be regulated via SRE. Of note, direct evidence only exists for SRE-dependent regulation of the Cav1 isoform e.g. [25]. However, when we searched 1 KB of DNA, in the 5′ flanking region of Cav3, for SRE binding protein consensus sequences, we identified 4 sites for rat Cav3 (−2651, −4401, −7201, −8351 base pair distances from the transcription start site, with a >85% binding probability) (http://www.cbrc.jp/research/db/TFSEARCH.html), providing some support for the concept of SRE control of Cav3 transcription in this species.

Manipulation of cellular cholesterol also affects Cav3 distribution in the cell [26], [27]. So next we looked specifically at Cav3 distribution within the membrane following sucrose density gradient fractionation. Statin treatment significantly reduced Cav3 levels in caveolar buoyant fractions 4 and 5, and increased levels in non-buoyant fractions 9–12 which represent non-cholesterol enriched membranes and cytosolic proteins (Fig. 2C). Thus, simvastatin reduces both cellular and caveolar Cav3, which is consistent with demonstrated effects on cholesterol (which binds Cav3 and regulates its expression/distribution).

Although the focus of our work is the muscle specific Cav3 isoform, some Cav1 is also found in the cardiac myocyte. We measured levels of Cav1 in cell lysates. There was significant variability in Cav1 expression between cell populations in the absence of statin treatment, and a trend (P>0.05) for lower Cav1 expression (normalised to GAPDH) in statin treated cells vs. controls (6.57±2 vs. 8.44±3.08; n = 4).

### Simvastatin reduces the density of caveolae

**Figure 3:**
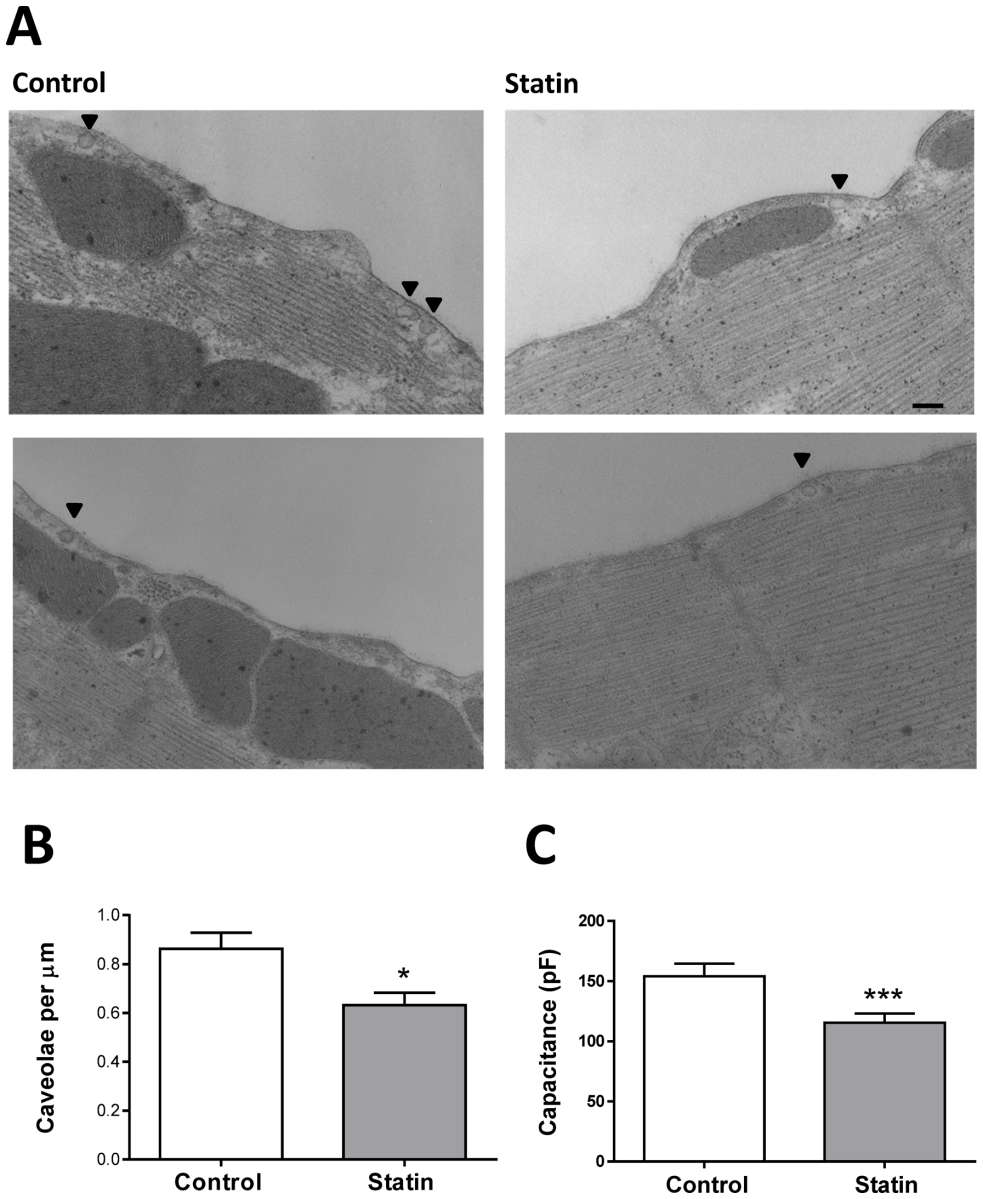
Simvastatin treatment reduces caveolar density. A. Representative membrane from control and simvastatin-treated cardiac myocytes. Arrows indicate caveolae. Scale bar represents 200 nm. B. Simvastatin treatment reduced the mean density of caveolae measured in ≈330 µm of membrane (n = 9 myocytes from 3 hearts). * P<0.05, Student's t-test. C. Simvastatin treatment reduced cell capacitance (n = 27–33 cells from 19 hearts). *** P<0.001 Mann Whitney Rank test.

Given that simvastatin depletes the myocyte membrane of both cholesterol and Cav3, the two essential components of caveolae, we predicted that this would cause a reduction in caveolar density. Representative electron micrographs of sections of membrane from control and statin-treated myocytes are shown in Figure 3. We observed a significant ≈30% reduction in caveolar density from 0.86±0.11 µm^−1^ membrane in controls to 0.63±0.08 µm^−1^ in treated myocytes (P<0.05). Interestingly, we also recorded a significant reduction in cell capacitance in statin-treated cells compared with controls (Fig 3C), which is consistent with reduced electrically-accessible membrane (i.e. ‘open’ caveolae, see [27]).

### Simvastatin modulates basal myocyte function

**Figure 4:**
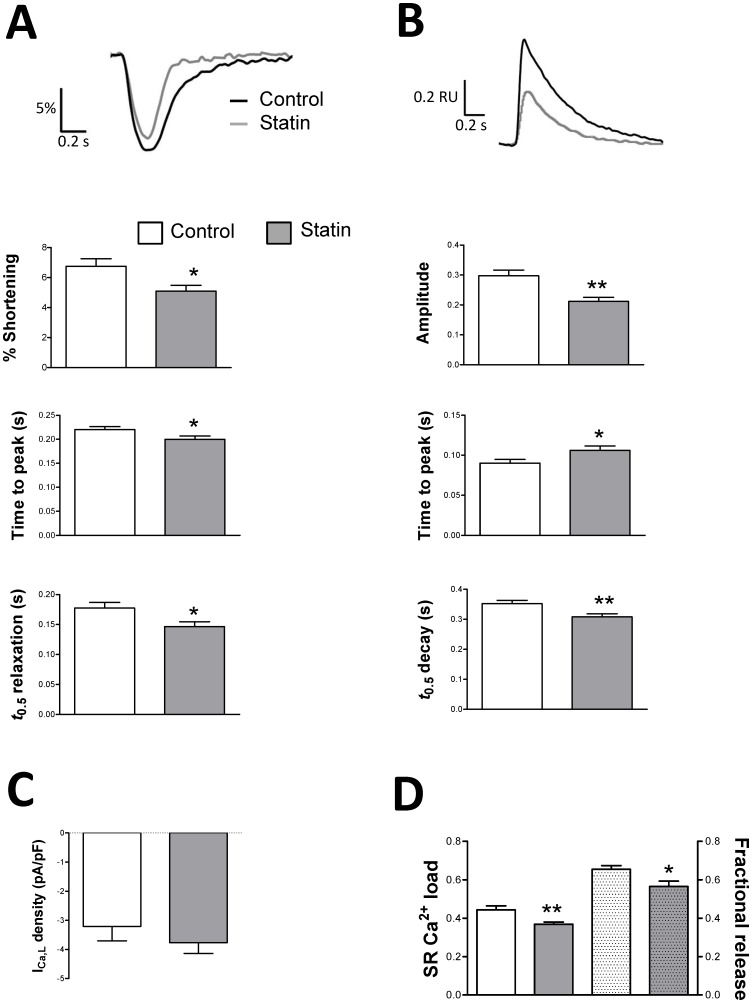
The effect of simvastatin treatment on shortening, [Ca^2+^]_i_ handling and *I*_Ca,L_ under basal conditions. A. Representative traces and mean data of amplitude and kinetics of shortening. Shortening amplitude is expressed as a % of resting cell length. There was no difference in resting cell length between groups (97±2 vs. 101±3 µm in control and statin-treated cells respectively). n = 33–41 myocytes from 5 hearts. B. Representative traces and mean data of amplitude and kinetics of the [Ca^2+^]i transient. Transient amplitude is expressed in fura-2 ratio units (RU). There was no difference in diastolic [Ca^2+^]i between groups (0.78±0.01 vs. 0.80±0.01 RU in control and statin-treated cells respectively). n = 33–41 cells from 5 hearts. C. Peak *I*Ca,L was measured following a step from −40 to 0 mV. n = 6–8 cells from 3 hearts. D. Sarcoplasmic reticulum (SR) Ca^2+^ load and fractional SR Ca^2+^ release. The left side of the panel shows the amplitude of [Ca^2+^]i transients (expressed as RU) induced by rapid application of 10 mM caffeine, an index of SR Ca^2+^ load. On the right, fractional release (the amplitude of the steady-state electrically-stimulated transient/caffeine-induced transient) is shown. n = 33–36 cells from 4 hearts. * P<0.05; ** P<0.01, Student's t-test.

Are statin-induced changes in cholesterol and Cav3 associated with changes in basal cardiac myocyte function? As shown in Figure 4, simvastatin treatment significantly reduced (P<0.05) [Ca^2+^]_i_ transient amplitude and shortening. Kinetics of [Ca^2+^]_i_ transients and contraction were also modulated by simvastatin. Most notably, we saw a positive lusitropic effect of statin treatment; statin-treated cells exhibited a significant reduction in time to half (*t*_0.5_) relaxation which was accompanied by a corresponding hastening of [Ca^2+^]_i_ transient decay (Fig. 4A,B). Simvastatin had no effect on the amplitude of *I*_Ca,L_ (Fig. 4C) suggesting that effects on [Ca^2+^]_i_ transient amplitude and contractility are due to alterations in sarcoplasmic reticulum (SR) function. Indeed, Figure 4D shows that simvastatin reduced (P<0.05) both SR Ca^2+^ load and fractional SR Ca^2+^ release indexed using caffeine. Simvastatin's effects on excitation-contraction coupling are similar to those reported following caveolae disruption with the cholesterol-depleting agent methyl-β-cyclodextrin (MBCD); reduced [Ca^2+^]_i_ transient amplitude and shortening in MBCD-treated cells was ascribed to reduced fractional SR Ca^2+^ release without any change in *I*_Ca,L_ [17].

The impact of simvastatin on basal contractility can be ascribed to inhibition of HMG CoA reductase because supplementation of simvastatin-containing culture medium with mevalonate (the product of HMG CoA reductase) significantly enhanced (P<0.001) basal shortening compared with simvastatin alone (6.3±0.7 vs. 4.0±0.3% of resting length; n = 34–40 cells) and shortening in myocytes cultured with simvastatin and mevalonate was not different (P>0.05) to that in control cells (6.8±0.5%; n = 41).

### Simvastatin enhances β2-AR responsiveness

**Figure 5:**
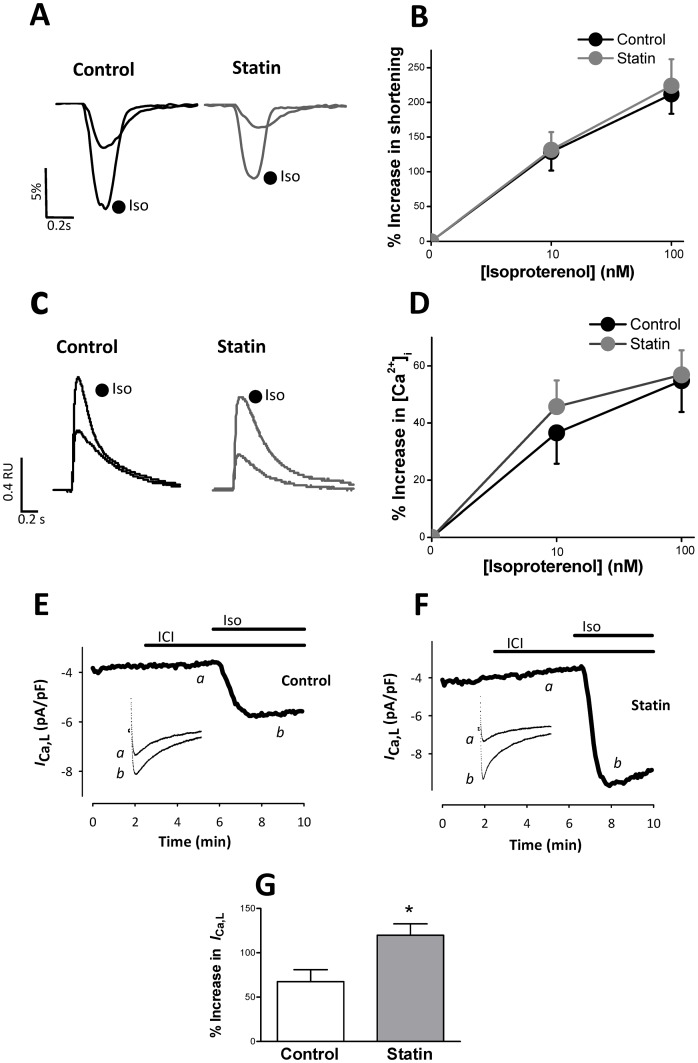
The effect of simvastatin treatment on the response to selective β1-AR stimulation. A, B. Representative traces and mean data showing the response of shortening (expressed as a % of resting cell length) to selective stimulation of β1-AR with 100 nM isoproterenol (Iso) in the presence of 100 nM ICI 118,551. C, D. Representative traces and mean data of the response of [Ca^2+^]i transient amplitude to selective β1-AR stimulation. Data are from 20–30 myocytes from 7 hearts. E, F, G. Representative traces and mean data of the response of *I*_Ca,L_ to selective stimulation of β1-AR with 10 nM Iso in the presence of ICI 118, 551 (ICI). n = 6–8 myocytes from 3 hearts. * P<0.05, Student's t-test.

**Figure 6:**
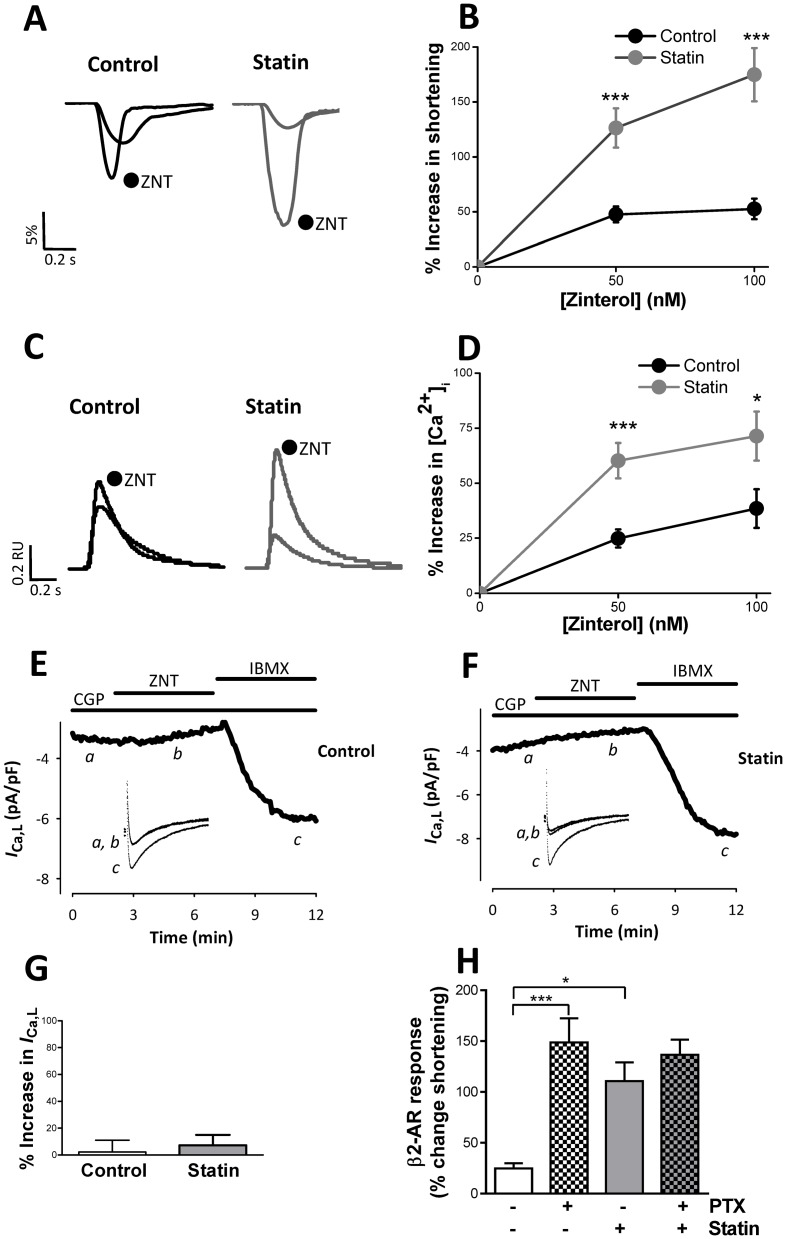
The effect of simvastatin treatment on the response to selective β2-AR stimulation. A, B. Representative traces and mean data of the response of shortening (expressed as a % of resting cell length) to selective stimulation of β2-AR with 100 nM zinterol (ZNT) in the presence of 10 nM CGP20712A. *** P<0.001 vs control. C, D. Representative traces and mean data of the response of [Ca^2+^]i transient amplitude to selective β2-AR stimulation. Data are from 24–30 myocytes from 7 hearts. * P<0.05; *** P<0.001 vs control. E, F, G. Representative traces and mean data of the response of *I*_Ca,L_ to selective stimulation of β2-AR with 100 nM ZNT in the presence of CGP20712A (CGP). Neither control nor simvastatin-treated myocytes showed a significant response of *I*_Ca,L_ to selective β2-AR stimulation (one sample t-test; data from 13–15 myocytes from 7–8 hearts), although in every case a robust *I*_Ca,L_ response to 100 µM IBMX was evoked. H. Bar graph shows the effect of pertussis toxin (PTX) treatment on the % change in shortening in response to β2-AR stimulation with 100 nM ZNT plus CGP. n = 25 myocytes from 5 hearts. * P<0.05; *** P<0.001, 2-way ANOVA.

Cholesterol-dependent disruption of caveolae with MBCD in the adult ventricular myocyte enhances β2-AR responsiveness and increases the sensitivity of functional responses to β1-AR stimulation [16], [17], [19]. To our knowledge, the only study to date to investigate the effect of statin treatment on β-AR signalling in the ventricular myocyte has shown reduced (non-selective) β-AR responsiveness in neonatal cardiac myocytes treated with atorvastatin *in vitro*; effects were ascribed to isoprenylation-dependent effects on Gαs [28]. Together this work suggests that simvastatin has the potential to modulate the way that the adult heart responds to β-AR stimulation through both cholesterol and isoprenoid-dependent mechanisms. Therefore we next determined the consequences of simvastatin treatment for the response to selective stimulation of β1- and β2-AR. There was no significant difference (P>0.05) between control and statin-treated myocytes in the response of shortening or [Ca^2+^]_i_ transient amplitude to β1-AR stimulation with 10 or 100 nM isoproterenol (in the presence of the β2-AR antagonist ICI; Fig. 5A-D). However, the response of *I*_Ca,L_ to 10 nM isoproterenol was significantly increased (P<0.05) by 80% in statin-treated cells (Fig. 5E-G). By contrast, statin treatment markedly enhanced the response of shortening and [Ca^2+^]_i_ transient amplitude to selective stimulation of the β2-AR with 50 and 100 nM zinterol (in the presence of the β1-AR antagonist CGP; Fig. 6A-D). We saw no significant *I*_Ca,L_ response to β2-AR stimulation in control cells (as we have reported previously [19]) (Fig. 6E). The statin-induced increase in β2-AR inotropic responsiveness could not be ascribed to changes in *I*_Ca,L_ (Fig. 6F,G). Caveolae compartmentalise cAMP-dependent signalling by facilitating β2-AR coupling to Gαi [19]. In order to test whether enhanced β2-AR responsiveness with statin treatment could likewise be attributed to effects on Gi, we compared the effect of abolishing Gi signalling with PTX in control and statin-treated cells. PTX enhances β2-AR inotropic responses in control cells (Fig. 6H) to a level comparable with that induced by statin treatment, and PTX had no impact (P>0.05) on β2-AR responses in statin-treated cells. Together these data suggest that statin treatment enhances β2-AR responses by restricting β2-Gi coupling.

In cells co-cultured with simvastatin and mevalonate, β2-AR responsiveness to 100 nM zinterol (with CGP) was significantly reduced (P<0.001) compared with cells cultured with simvastatin alone (6±3 vs. 72±14% increase in shortening over baseline; n = 32–37 cells). These data are consistent with enhanced β2-AR responsiveness being mediated through simvastatin inhibition of HMG CoA reductase.

**Figure 7:**
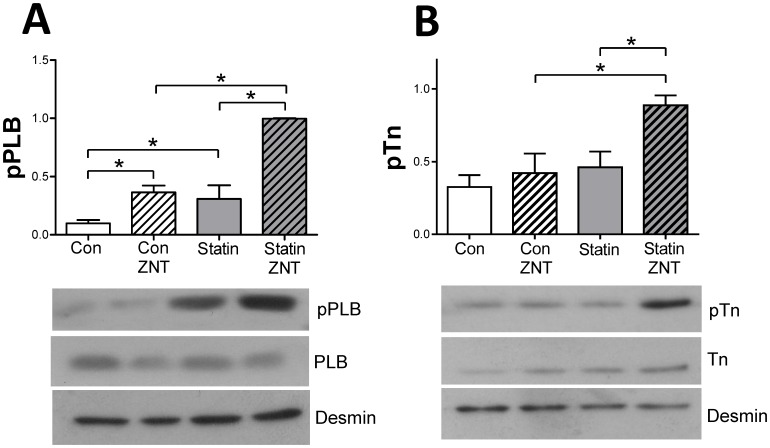
Simvastatin treatment modulates phosphorylation of PLB and TnI following β2-AR stimulation. A. Representative immunoblots and mean data showing levels of Ser^16^ phosphorylated phospholamban (pPLB) under basal conditions and following β2-AR stimulation with 100 nM zinterol (ZNT) +10 nM CGP20712A. B. Representative immunoblots and mean data showing levels of Ser^23/24^ phosphorylated troponin I (pTnI) under basal conditions and following β2-AR stimulation. Simvastatin had no significant effect (P>0.05) on expression of PLB or TnI. All phosphorylated protein data are normalised to desmin expression. n = 4 hearts. * P<0.05, 2-way ANOVA.

We have previously shown that increased β2-AR responsiveness following caveolae disruption is due to enhanced phosphorylation of the SR protein phospholamban (PLB) at Ser^16^, the PKA site [19]. Figure 7A shows levels of Ser^16^ phosphorylated PLB (pPLB) in control and statin-treated samples under basal conditions and following selective β2-AR stimulation with 100 nM zinterol. Interestingly, under basal conditions (i.e. in the absence of β2-AR stimulation) we saw a significant 300% increase (P<0.05) of pPLB in statin-treated cells compared with controls, which could explain the faster transient decay and relaxation kinetics in these cells (see Fig. 4A,B). This was seen in the absence of any difference (P>0.05) in expression of PLB (1.95±0.33 vs. 2.20±0.21), SERCA2a (2.85±0.26 vs. 2.23±0.27) or in the PLB/SERCA ratio (0.73±0.17 vs. 1.03 vs. 0.17) between control and statin-treated cells respectively (values normalised to GAPDH; n = 4 hearts). Both groups showed a significant change (P<0.05) in pPLB with β2-AR stimulation, but the increase in pPLB was greater in statin treated cells. Troponin I (TnI) is also phosphorylated by PKA at Ser^23/24^. There was no difference in basal pTnI between groups, and β2-AR stimulation did not alter pTnI in control cells. However, in statin-treated cells, β2-AR stimulation resulted in a 92% increase (P<0.05) in pTnI over basal levels (Fig. 7B). Together these data suggest that statin treatment changes the amplitude and spatial characteristics of cAMP-dependent signalling following β2-AR stimulation.

It is worth highlighting that ventricular myocytes cultured for 2 days show differences in their response to β2-AR stimulation (notably increases in pPLB, associated with significant lusitropy which are absent in non-cultured cells) [19]. This suggests a change in the normal local/global pattern of cAMP-dependent signalling. Thus, although we used culture conditions which best preserve myocyte morphology and function, it is impossible to fully preserve the phenotype of the freshly dissociated myocyte.

### Simvastatin increases NO production

**Figure 8:**
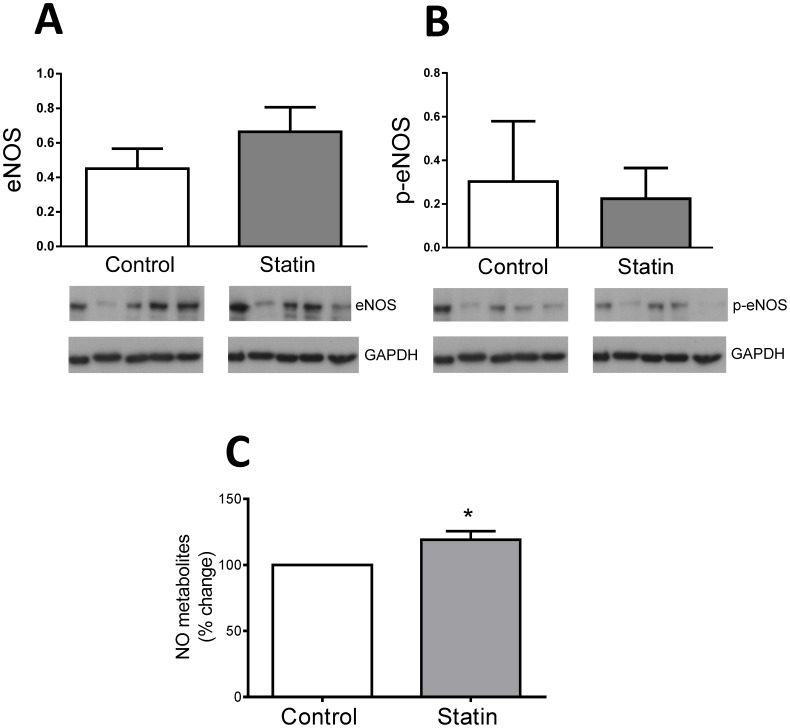
The effect of simvastatin on eNOS and NO production. A. Total eNOS expression normalised to GAPDH in control and simvastatin-treated cells. B. Ser^1177^-phosphorylated eNOS normalised to GAPDH in control and simvastatin-treated cells. Data are from cells from n = 5 hearts. C. Medium was collected from myocytes cultured for 48 h in the presence or absence of simvastatin. NO metabolites (nitrate/nitrite) were measured by a fluorimetric assay. Data are [nitrite + nitrate] normalised to values in paired control group (cells from n = 6 hearts). * P<0.05, Student's t-test.

Statins have been shown to modulate NO production in a variety of cell types [9], [10], [29]. NO modulates basal and sympathetic regulation of myocyte function [30], [31] therefore NO effects may contribute to observed changes in basal and β-AR stimulated function following statin treatment. Endothelial NOS (eNOS) is the primary constitutive NOS in the myocardium, expressed in both endothelial cells and the cardiac myocyte itself [32]. Therefore we measured expression of eNOS and its phosphorylation at the Ser^1177^ Akt site (p-eNOS) in myocyte lysates. We were unable to detect nNOS expression in our myocyte lysate, likely because of very low expression of this protein. There was no significant difference (P>0.05) in eNOS expression or in the amount of p-eNOS (i.e. p-eNOS normalised to GAPDH) between control and statin-treated cells (Fig. 8A,B). A key regulator of eNOS (and nNOS) is caveolin, which exerts a tonic inhibitory influence on NOS activity [33], [34], therefore NO production can be regulated in the absence of changes in eNOS expression or phosphorylation. We measured NO metabolites (nitrates and nitrites) as an index of NO in medium from myocytes cultured for 2 days in the presence or absence of simvastatin. In statin-treated cells NO metabolites were ≈20% higher than in controls (P<0.05; Fig. 8C), consistent with enhanced NOS activity secondary to reduced Cav3 expression.

### The contribution of cholesterol and isoprenoids to simvastatin's effects

**Figure 9:**
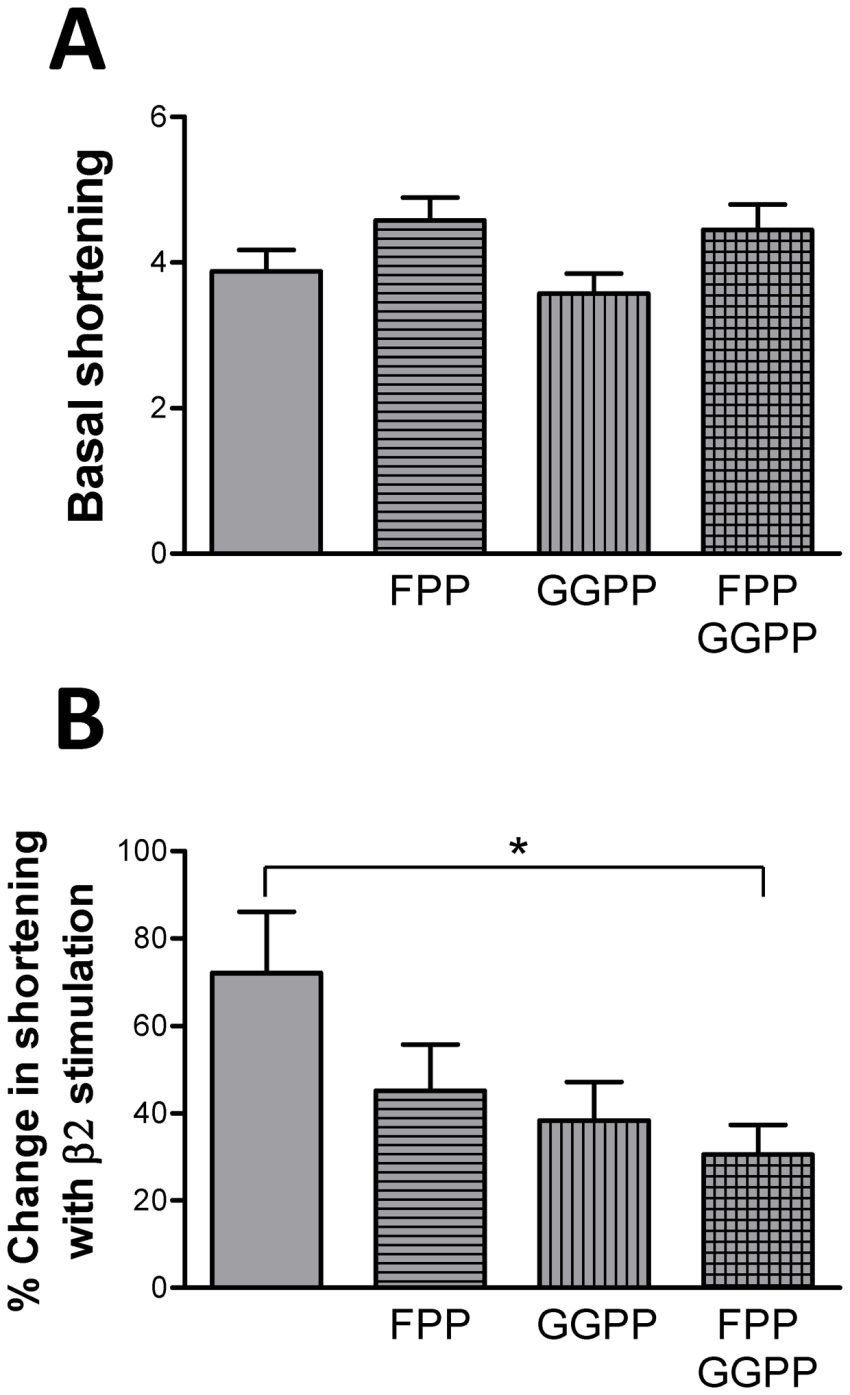
Isoprenoid dependence of simvastatin effects on basal function and the response to β2-AR stimulation. Myocytes were cultured with simvastatin in the presence of farnesyl pyrophosphate (FPP), and/or geranylgeranyl pyrophosphate (GGPP). A. Basal shortening (as a % of resting cell length). B. % change in shortening in response to β2-AR stimulation with 100 nM zinterol plus 10 nM CGP20712A. n = 32–38 myocytes from 7 hearts * P<0.05, 1-way ANOVA.

In general, the effects of simvastatin on basal and β-AR stimulated adult myocyte function are very similar to those we have reported previously when caveolae are disrupted with MBCD, suggesting that simvastatin is working through cholesterol-dependent (caveolar) pathways. However, given the body of data that show isoprenoid-dependent statin effects in neonatal cardiac myocyte and non-cardiac cells, we looked at the possible contribution of isoprenoids to the observed functional effects. Basal shortening and the inotropic response to β2-AR stimulation were measured in cells cultured with statins in the presence of FPP and/or GGPP. Isoprenoid supplementation did not increase (P>0.05) basal shortening in statin-treated cells (Fig. 9A), thereby ruling out a role for protein prenylation in depressed basal myocyte function. There was a trend for reduced β2-AR responsiveness when statin-treated myocytes were cultured in the presence of either FPP or GGPP but these differences were not significant (P>0.05; Fig. 9B). However, when FPP and GGPP were used together, a significant reduction (P<0.05) in β2 responsiveness was observed. This suggests that isoprenoid-dependent effects may contribute to simvastatin's ability to enhance β2-AR responsiveness.

## Discussion

Statin treatment of cultured myocytes is an excellent experimental model in which the concentration of drug and duration of treatment can be exactly controlled, and contribution from non-myocytes excluded. In the present study we have treated cells for 2 days with 10 µM simvastatin, a concentration which is widely used *in vitro* and shows no evidence of toxicity. Our chosen regime results in an identical depletion of cellular cholesterol to that seen following a 5 day treatment with 1 µM simvastatin, yet allows us to best preserve the adult myocyte phenotype.

Until recently, very little was known about cholesterol homeostasis in the cardiac muscle cell [35]. However, recent work has demonstrated that (neonatal) ventricular myocytes control cholesterol predominantly through cholesterol efflux rather than internalisation of native or acetylated LDL [11]. These authors focused on the ABC transporters as cholesterol efflux pathways, however caveolin has also been linked with cholesterol efflux to HDL [36], [37]. Here we show further evidence for a cholesterol-dependent control of efflux pathways. Inhibition of *de novo* cholesterol synthesis by simvastatin reduced cellular and caveolar cholesterol in the adult cardiac myocyte and this was, in turn, associated with a significant depletion of cellular and caveolar Cav3. Ultimately this change could act to preserve cellular cholesterol by reducing efflux. However, decreased caveolin expression has many additional consequences for cardiac cell function.

Caveolar density was reduced in simvastatin-treated myocytes, as we would predict from reduced cellular and caveolar levels of cholesterol and Cav3. Although myocyte cholesterol levels would tend to normalise with statin treatment *in vivo*, we predict that the maintained decrease in cellular caveolin required to effect this normalisation of cholesterol would be sufficient to reduce the number of caveolae even in the presence of normal cellular cholesterol, since caveolin is required for the presence of caveolae e.g. [14].

Simvastatin-disruption of caveolae was associated with 2 fundamental changes in cardiac myocyte contractility: a decrease in the degree of shortening and an increase in the rate of relaxation. Both could be explained by corresponding changes in the [Ca^2+^]_i_ transient. Our data suggest that the reduction in amplitude of [Ca^2+^]_i_ transient is due to reduced Ca^2+^ -induced Ca^2+^ release secondary to decreased SR Ca^2+^ load. The hastening of Ca^2+^ transient decay is consistent with the 3-fold increase in pPLB (without changes in total PLB or PLB/SERCA2a ratio) measured in statin-treated cells. However, in the absence of other changes in Ca^2+^ handling processes, the predicted effect of enhancing SERCA activity by increased pPLB would be an increase in SR Ca^2+^ content, [Ca^2+^]_i_ transient amplitude and contractility [38], which is opposite to the observed effect. Therefore additional statin effects must be responsible for maintaining SR Ca^2+^ load at a lower level (perhaps via NO-induced SR Ca^2+^ leak, see below). A previous study has reported increased SERCA, but not PLB, expression using a similar *in vitro* treatment regime with 10 µM simvastatin, however these experiments were performed with neonatal ventricular myocytes which differ structurally and functionally from their adult counterparts [39].

Here we report no impact of statin treatment on the response to β1-AR stimulation with 100 nM isoproterenol, but increased *I*_Ca,L_ and a trend for increased [Ca^2+^]_i_ transient responses to 10 nM isoproterenol. This is consistent with enhanced sensitivity of functional responses to β1-AR stimulation, comparable with data obtained using MBCD to disrupt myocyte caveolae [16]. Increased adenylyl cyclase activity, by removal of the normal inhibitory influence of Cav3 [40], may contribute to this effect. The disparity between consequences of statin treatment for *I*_Ca,L_ and contraction suggests there may be some selective effect of statin treatment on extra-junctional L type Ca^2+^ channels which are not involved in excitation-contraction coupling (e.g. see [41]). We also report a marked enhancement of the inotropic response to β2-AR stimulation in statin-treated cells which mimics that seen following MBCD treatment [19]. β2-ARs couple sequentially to Gs and Gi proteins and prevention of Gi coupling enhances β2-AR responsiveness [17], [42]. Statin effects are consistent with uncoupling of β2-AR and Gi, as simvastatin treatment mimics abolition of Gi signalling by PTX, and statin-treated cells are unresponsive to PTX. Further evidence for the impact of simvastatin on Gi comes from the apparent conversion of a sarcolemmal/sarcoplasmic reticular confined cAMP-dependent β2-AR signal in control cells to a global signal which targets the myofilament protein TnI in statin-treated cells. β2-AR induced phosphorylation of PLB was also enhanced by statin treatment. Although myocytes cultured for 2 days do show some differences in the sensitivity and spatial characteristics of cAMP-dependent signalling when compared with non-cultured cells, empirically the effects of statin treatment on protein phosphorylation are equivalent to that induced by PTX [43].

What is responsible for the sequelae of statin treatment? We have shown that altered basal and β-AR stimulated function is due to simvastatin inhibition of HMG CoA reductase inhibition. Altered basal contractility cannot be normalised by supplementation of culture medium with FPP and GGPP suggesting that this is an isoprenoid-independent mechanism, whereas supplementation with FPP and GGPP together does partially revert statin potentiation of β2-AR responses, suggesting a role for protein prenylation in this. The fact that both FPP and GGPP supplementation is required to partially reverse statin-induced changes in β2-AR responsiveness suggests a complex pathway. The individual roles for farnesylation and geranylgeranylation require clarification.

One key mechanism which has been proposed for statin pleiotropy is increased NO production. Statins can enhance NO production via depletion of cellular isoprenoids (increased expression and Akt-dependent phosphorylation of eNOS) [8], [9] and caveolin (increased activity of eNOS and nNOS) [12], [34], [44]. In the intact myocardium, eNOS is expressed in both endothelial cells and cardiac myocytes, with higher expression in the former [32], supporting the view of a strong paracrine influence of endothelial cell NO on myocyte function. eNOS expression and Ser^1177^ phosphorylation were not significantly different between control and statin-treated groups suggesting a minimal role for isoprenoid-modulation of eNOS activity in the cardiac cell. However, our novel finding of decreased caveolin expression in cardiac cells following statin treatment can account for enhanced NO production.

It is difficult to interpret the consequences of statin treatment for cardiac myocyte function observed in the present study simply in terms of increased NO production, in part because the concentration and location of NO are key factors which determine its effects (eNOS and nNOS show different subcellular distribution) and in part because there is no clear consensus from different laboratories about the effect of NO derived from different sources. Data from NOS knockout mice suggest that eNOS derived NO has little effect on basal myocyte contractility, whereas nNOS-derived NO decreases or increases contractility whilst hastening relaxation (see [45], [46]). The latter phenomena mimic the impact of statins on basal function reported here. However, the underlying causes of altered amplitude and kinetics of contraction differ in some respects between statin treatment and the effects of nNOS-derived NO. Although nNOS-derived NO may hasten relaxation by increasing pPLB (secondary to altered phosphatase activity) [47], which is consistent with our own data in statin-treated cells, decreased *I*_Ca,L_ has been identified as one mechanism which subtends impaired contractility as a result of nNOS-derived NO [48] yet we detected no change in *I*_Ca,L_ following statin treatment. Furthermore, it is necessary to account for the observed decrease in SR Ca^2+^ load with simvastatin treatment, seen despite elevated pPLB. One possibility is an increase in SR Ca^2+^ leak via NO-dependent *S*-nitrosylation of RyR [49].

**Figure 10:**
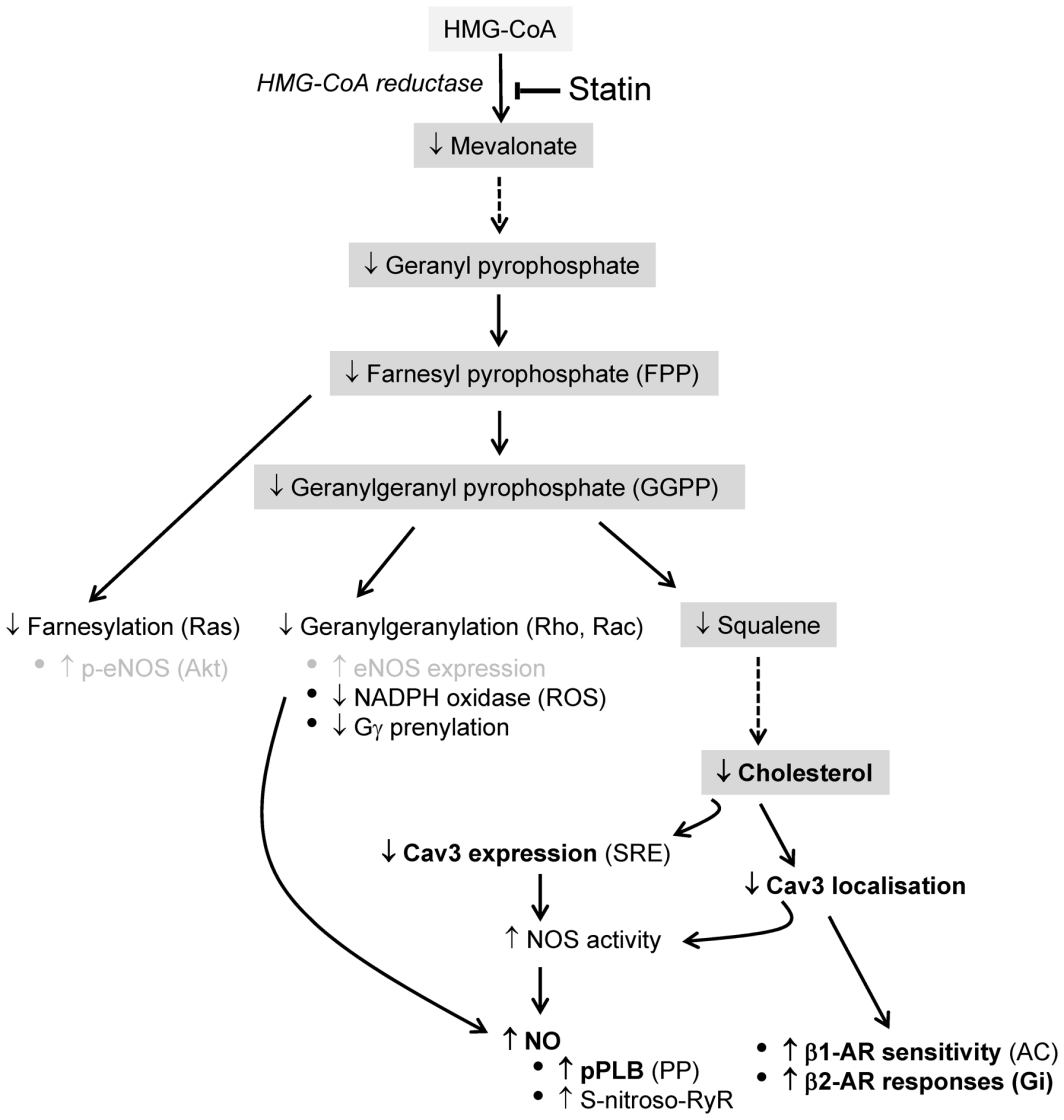
Statin effects on the cardiac myocyte. The main cholesterol synthesis pathway is highlighted in grey. The impact of statin inhibition of HMG-CoA reductase, the rate limiting step of cholesterol synthesis, is shown. Statins limit production of cholesterol and isoprenoid intermediates (FPP, GGPP). The latter are responsible for post-translational modification of a number of proteins including those of the small G protein family (Ras, Rho, Rac). The potential roles of isoprenoid- and cholesterol-dependent changes in modulating cardiac myocyte function are listed. From the present study, **bold text** indicates established effects, whereas grey text denotes effects which have been eliminated. ROS reactive oxygen species (which reduce bioavailable NO); PP phosphatase; AC adenylyl cyclase. See Discussion for detailed mechanisms and references.

In terms of NO effects on β-AR responsiveness, it is generally agreed that eNOS-derived NO limits the response to non-selective β-AR stimulation whereas nNOS-derived NO effects are more diverse (see [50], [51]). The marked enhancement of β2-AR responses in the absence of a change in β1-AR inotropic responses with statin treatment is not consistent with effects mediated via NO. Instead, we argue that the impact of statin treatment on β-AR responsiveness is due directly to disruption of caveolae. Statin effects mirror MBCD-mediated disruption of caveolae which alters the amplitude and spread of cAMP-dependent signalling by limiting cAMP production and promoting phosphatase activity. We suggest that this occurs because the β2-AR is prevented from making its usual coupling with Gαi [19]. Importantly for the present study, effects of MBCD on β2-AR responsiveness are mimicked by inhibiting Cav3 interactions within the myocyte [19]. This implies that changes in caveolin alone (i.e. in the absence of depletion of cholesterol) heighten the β2-AR cAMP signal. Therefore, even after decreased expression of Cav3 promotes normalisation of cellular cholesterol, statin-dependent effects on β2-AR responsiveness will be maintained. Figure 10 summarises potential pathways by which statins may impact on cardiac myocyte function.

We are aware of the limitations of this work to the clinical situation where statins are taken long-term *in vivo*; our aim was to isolate the direct effects of statins on the cardiac myocyte. *In vivo*, contributions from statin effects on other cells of the myocardium (particularly endothelial cells, a major source of eNOS derived NO) will be of significance, as well as the long-term compensatory changes in cholesterol homeostasis. So how do our data relate to what is known about myocardial function in people taking statins? There are several reports of improved cardiac function (measured using echocardiography) following statin treatment [52]–[54]. However, these were in hypercholesterolaemic patients, so it is impossible to differentiate between improvement of serum cholesterol and direct effects on the myocardium. Interestingly, a recent study has shown that in *normocholesterolaemic* subjects, statin treatment improves myocardial function indexed by strain imaging [55].

On balance, the effects of statins that we observe in this study are unlikely to compromise cardiac function in healthy individuals. In patients with heart failure, the observed direct effects of statin treatment on the cardiac myocyte are likely to be of benefit. A mild reduction in basal contractility may, similar to β-blockers, improve cardiac function by reducing myocardial energy expenditure. Increased myocardial relaxation will improve diastolic filling. In heart failure, the ratio of β1/β2-AR decreases, and Gi protein increases [56]. Our findings suggest that statin treatment could enhance β-2 AR responsiveness by limiting Gi coupling, which may be beneficial in maintaining the contractile reserve of the heart. On a cautionary note, this may occur at the expense of the cardioprotective, anti-apoptotic Gi signalling arm of the β2-AR [57], [58].

Our study presents a fundamental view of the effects of statins on the cardiac myocyte by treating myocytes *in vitro*. This approach has allowed us to show for the first time that statins can modulate myocyte function via cholesterol-dependent effects on caveolin expression. Furthermore, as caveolin isoforms are ubiquitously expressed and have myriad tissue-specific functions, the impact of statin-dependent changes in caveolin is likely to have many other functional sequelae.

## Methods

### Ethics statement

All animal experimentation was carried out in accordance with the Directive 2010/63/EU of the European Parliament. Adult male Wistar rats were group housed with 12 h light/dark cycles and free access to food and water. Animals were used to harvest tissue only and were sacrificed according to a Schedule 1 method of the Animal (Scientific Procedures) Act of 1986 at a designated establishment.

### Myocyte isolation and culture

Ventricular myocytes were isolated enzymatically from hearts of rats (250–280 g) according to the method described by Calaghan et al. [59]. Cells were cultured using a method based on that developed by [60] which is designed to minimise changes in the adult myocyte phenotype during the culture period. In brief, immediately after isolation, myocytes were plated on laminin and cultured in serum-free Medium 199 with 5 mM creatine, 5 mM taurine, 2 mM Na pyruvate, 2 mM L-carnitine plus 0.1 µM insulin and 2 µg/ml Primocin (Amaxa). Medium was supplemented with 10 µM sodium simvastatin (Merck Chemicals Ltd) or vehicle (0.1% DMSO) for 48 h. As medium lacked LDL and cholesterol acceptors (apolipoproteins, HDL), any effects of HMG CoA inhibition on cholesterol influx or efflux pathways (see Introduction) were minimised. Only adherent (viable) myocytes were used for subsequent biochemical or functional assays.

### Sucrose density gradient fractionation

Myocytes were fractionated using detergent-free methods as described previously [18]. Adherent myocytes were scraped from culture wells using 500 mM Na_2_CO_3_ (pH 11.0) containing 0.5 mM EDTA and 1% protease inhibitor cocktail (Sigma). Samples were homogenised (Ultra-Turrax T8; Ika), then sonicated (Vibra Cell; Sonics) 3 times each for 20 s at full power. Approximately 2 ml of homogenate was mixed with an equal volume of 90% sucrose in MES-buffered saline (25 mM MES, 150 mM NaCl, 2 mM EDTA, pH 6.5) to form a 45% sucrose solution. A discontinuous sucrose gradient was created by layering on to this a further 4 ml each of 35% and 5% sucrose solution (MES-buffered saline with 250 mM Na_2_CO_3_). Gradients were centrifuged for 17 h at 280,000 *g* (Beckman SW40Ti rotor) at 4°C. A total of 12 fractions (each 1 ml) were collected following fractionation.

### Cholesterol measurement

Free cholesterol distribution was imaged in myocytes fixed with 2% paraformaldehyde for 1 h at room temperature, and stained with filipin solution (50 µg.ml^−1^ with 4% donkey serum in phosphate-buffered saline) for 1 h at 37°C (see [16], [61]). Sections of approximately 2 µm thickness were acquired by confocal laser microscopy (Zeiss LSM 510) with excitation at 364 nm and detection at 385–470 nm. Identical confocal settings were used to acquire images of control and statin-treated myocytes. Whole-cell fluorescence was quantified using ImageJ software. Total cholesterol was measured in cell homogenates before and after fractionation using the Amplex Red assay (Invitrogen). For cholesterol levels in unfractionated samples, cholesterol was normalised to the protein content of each sample. For fractionated samples, to allow comparison of cholesterol distribution, proteins levels for all samples were normalised prior to fractionation, and cholesterol measured in equivalent fraction volumes.

### Immunocytochemistry

The distribution of Cav3 (the muscle-specific isoform) in fixed, triton-permeabilised myocytes was imaged by immunocytochemistry as described in [17]. In triton-permeabilised cells, non-tethered proteins will be dialysed during sample preparation [62], therefore this method will preferentially label membrane-tethered Cav3. Identical confocal settings were used to acquire images of control and statin-treated myocytes.

### Western blotting

Protein expression in cell lysates before and after fractionation was measured by Western blotting as described in [59]. Desmin and GAPDH were used to ensure equal protein loading of cell lysate samples; statin treatment had no effect (P>0.05) on expression of either protein (desmin 6.7±0.59 vs 7.03±1.24 [n = 4] and GAPDH 6.26±0.23 vs 6.16±0.27 [n = 5] arbitrary units, AU, for control and statin-treated cells respectively). For fractionated samples, equal volumes of fractions were loaded onto SDS-gels and band density normalised to the sum of the band density in all fractions.

### Electron Microscopy

Myocytes were fixed overnight with 2.5% glutaraldehyde, post-fixed using 1% osmium tetroxide and dehydrated through an ethanol series. Myocytes were embedded in araldite, sectioned and stained with 20% uranyl acetate (30 min) followed by Reynolds lead citrate (30 min). Sections were visualised using a JEOL 1200 transmission electron microscope. Caveolae were defined as either 50–150 nm flask-shaped invaginations in the surface membrane or sealed circular vesicles of the same size within 300 nm of the sarcolemma [27].

### Shortening and [Ca^2+^]_i_

Shortening and [Ca^2+^]_i_ were measured in fura-2 loaded myocytes field-stimulated at 0.5 Hz (22–24°C) in HEPES-based physiological solution containing (mM): NaCl 137; KCl 5.4; NaH_2_PO_4_ 0.33; MgCl_2_ 0.5; HEPES 5; glucose 5.5; CaCl_2_ 1 (pH 7.4) (Cairn Research Optoscan monochromator; Ionoptix Cell Contractility system). Parameters were measured under basal conditions and following selective stimulation of β1 or β2 ARs. Recordings were taken at the peak steady-state response (≈5 min) following application of agonist.

In order to assess sarcoplasmic reticular (SR) Ca^2+^ load and fractional SR ^Ca2+^ release, electrically-stimulated Ca^2+^ transients were recorded at steady state, stimulation was switched off and caffeine (10 mM) was applied 10 s later via a local perfusion device. SR Ca^2+^ load was indexed as the amplitude of the caffeine-stimulated Ca^2+^ transient. Fractional SR Ca^2+^ release was calculated from the ratio of electrically-stimulated to caffeine-stimulated Ca^2+^ transient amplitude.

### Electrophysiology

*I*_Ca,L_ was measured by voltage clamp, as described by [16]. Microelectrode resistances were between 1 and 2 MΩ when filled with intracellular solution containing (in mM) CsCl 130, TEA-Cl 20, EGTA 5, MgATP 5, TrisGTP 0.06, and HEPES 5 (pH 7.2) Cells were clamped at a holding potential of −80 mV. A 50 ms pre-pulse to −40 mV was used to inactivate *I*_Na_, followed by 100 ms test pulse to 0 mV to activate *I*_Ca,L_. Changes in *I*_Ca,L_ magnitude were monitored by repeating this protocol once every 5 s.

### Drug treatments

β1-AR stimulation was achieved with 10 and 100 nM isoproterenol in the presence of the β2 antagonist ICI 118,551 (ICI; 100 nM). Selective β2-AR stimulation was achieved with 50 and 100 nM zinterol (Tocris) in the presence of the β1 antagonist CGP20712A (CGP; 10 nM). Myocyte populations used to measure phospho-protein responses were field-stimulated during β-AR stimulation. At 5 min, perfusion buffer was rapidly aspirated and replaced with Laemmli sample buffer containing protease inhibitor cocktail (Roche) and phosphatase inhibitor cocktail (Thermo Fisher Scientific).

For experiments using pertussis toxin (PTX) to disable Gαi, cells were treated with 1.5 µg.ml^−1^ PTX for 3 h at 37°C. To determine the contribution of HMG CoA reductase inhibition and depletion of isoprenoids to basal and β-AR responses, mevalonate (100 µM), FPP and/or GGPP (10 µM) were added to statin-containing medium at the start of the 48 h culture period. For these protocols, shortening was measured in the absence of fura-loading.

### Measurement of NO metabolites

Quantification of nitrite and nitrate levels in culture medium was achieved using a method outlined by Nussler *et al*. [63]. Briefly, isolated myocytes from each heart were split into two populations and cultured at equal density. Samples of culture medium were taken after 48 h and ultra-filtrated using Centrisart 1 columns with a molecular weight cut-off of 10 kDa (Sartorius #12329E). Nitrate pools from resulting ultra-filtrates were converted to nitrite with nitrate reductase and the total nitrite concentration of each sample was determined using 2,3-diaminonapthalene, as described [63].

### Reagent suppliers

All reagents were from Sigma Chemical Co. unless otherwise stated. Antibodies used for Western blotting/immunocytochemistry: Cav 3 #610420 (1∶2500/1∶100), Cav1 #610406 (1∶2500), eNOS #610296 (1∶1000), Ser^1177^ phosphorylated eNOS # 612392 (1∶1000) (BD Biosciences), phospholamban #A010-14 (1∶5000), Ser^16^ phosphorylated PLB #A010-12 (1∶2000), SERCA #A010-23 (1∶2000) (Badrilla), TnI #4002 (1∶1500), Ser^22,23^ phosphorylated TnI #4004 (1∶1500) (Cell Signaling Technology), desmin #ab8592 (1∶1000) (Abcam), GAPDH #G9545 (1∶100,000) (Sigma).

### Statistics

Results are expressed as mean ± S.E.M of *n* observations. Where experiments were performed on individual cells, these were from at least 3 different hearts. Statistical analysis was performed using the Student's t-test, Mann-Whitney Rank test, one way- or two way-ANOVA (with post-hoc analysis by the Tukey test), as appropriate.
